# Organizing medical oncology care at a regional level and its subsequent impact on the quality of early breast cancer management: a before-after study

**DOI:** 10.1186/1472-6963-14-326

**Published:** 2014-07-28

**Authors:** Aline Voidey, Xavier Pivot, Anne-Sophie Woronoff, Gilles Nallet, Laurent Cals, Francis Schwetterle, Samuel Limat

**Affiliations:** 1 Department of Pharmacy, Jean Minjoz University Hospital, 2 boulevard Fleming, 25000 Besançon, France; 2 Department of Medical Oncology, Jean Minjoz University Hospital, 2 boulevard Fleming, 25000 Besançon, France; 3 Doubs and Belfort Territory Cancer Registry, Jean Minjoz University Hospital, 2 boulevard Fleming, 25000 Besançon, France; 4 IRFC-FC, Jean Minjoz University Hospital, 2 boulevard Fleming, 25000 Besançon, France; 5 Department of Medical Oncology, Belfort-Montbéliard Hospital, 14 rue de Mulhouse, 90016 Belfort, France; 6 Department of Gynecology, Lons le Saunier Hospital, 55 rue du Dr Jean-Michel, 39016 Lons-Le-Saunier, France

**Keywords:** Breast cancer, Quality measurement, Regional organization, Medical management, Economic evaluation

## Abstract

**Background:**

One of the main measures of the French national cancer plan is to encourage physicians to work collectively, and to minimize territorial inequities in access to care by rethinking the geographical distribution of oncologists. For this reason, cancer care services are currently being reorganized at national level. A new infrastructure for multidisciplinary cancer care delivery has been put in place in our region. Patients can receive multidisciplinary health care services nearer their homes, thanks to a mobile team of oncologists. The objective of our study was to assess, using a quality approach, the impact on medical management and on the costs of treating early breast cancer, of the new regional structure for cancer care delivery.

**Methods:**

Before-and-after study performed from 2007 to 2010, including patients treated for early breast cancer in three hospitals in the region of Franche-Comté in Eastern France. The main outcome measures were quality criteria, namely delayed treatment (>12 weeks), dose-intensity and assessment of adjuvant chemotherapy. Other outcomes were 24-month progression-free survival (PFS) and economic evaluation.

**Results:**

This study included 667 patients. The rate of chemotherapy tended to decrease, but not significantly (49.3% before versus 42.2% after, p=0.07), while the use of taxanes increased by 38% across all centres (59.6% before versus 98.0% after, p < 0.0001). There was a non-significant reduction in the time between surgery and adjuvant chemotherapy (6.0 ± 3.0 weeks before versus 5.6 ± 3.6 weeks after, p=0.11). Dose-dense chemotherapy improved slightly, albeit non significantly (86.3% versus 91.1% p=0.22) and time to treatment tended to decrease. The new regional infrastructure did not change 24-month PFS, which remained at about 96%. The average cost of treatment was estimated at €7000, with no difference between the two periods.

**Conclusions:**

Despite a shortage of oncologists, the new organization put in place in our region for the provision of care for early breast cancer makes it possible to maintain local community-based treatment, without negative economic consequences. This new structure for cancer care delivery offers cancer services of similar quality with no modification of 24-month PFS in early breast cancer.

## Background

Cancer incidence rates are increasing worldwide, with more people being affected each year (14.1 million in 2012) [1]. It is the leading cause of mortality in France (158,893 deaths in 2011) [2]. These data illustrate why cancer is considered as a public health priority. The complexity of cancer management has compelled the French government to introduce new measures for the provision of care. Through two successive national cancer plans (2003–2007 and 2009– 2013), France has shown its determination to fight against cancer. These plans aim to tackle the cancer problem at a national level. One of the main goals of these plans is to ensure efficient medical management. The 2009–2013 cancer plan aimed to pursue the goals of quality and organization of care laid down in the first plan between 2003 and 2007. Effective coordination of all health professionals is necessary to promote high quality and equitable services to all cancer patients. The national cancer plan emphasizes the important role of close multidisciplinary collaboration in improving the quality of healthcare. Moreover, access to innovative therapies, particularly clinical trials, is also essential in the fight against this disease. Patients need to benefit from the most recently developed treatments.

France is confronted with many demographic challenges. Given that cancer will continue to represent a major cause of morbidity and mortality in the coming decades, the relative shortage of qualified medical oncologists in France is worrying. Skilled medical practitioners are becoming scarce, and the shortage of oncologists has created disparities in the delivery of cancer care between the various regions of France, and this in turn impacts on quality of care. A major measure of the national cancer plan is to favor collective work, and to minimize territorial inequities in access to care by revising the geographical distribution of oncologists. To this end, cancer care services are being reorganized at a national level. French administrative regions have been encouraged to revise the distribution of cancer care services in order to ensure equal access to cancer care for all citizens, regardless of where they live. Furthermore, auxiliary services such as medical transport and home care also need to be improved to ensure seamless continuity of care, even after discharge from cancer centres.

To this end, a new regional infrastructure to coordinate a multidisciplinary approach to cancer treatment has been put in place. Since 2009, the *Institut Régional fédératif du cancer en Franche-Comté* (IRFC-FC, federative regional institute for cancer of Franche-Comté) has been the regional oncology reference centre for the Franche-Comté region (an administrative region in Eastern France with a population of approximately 1.2 million). This organization received the approval of French national authorities and the *Institut National du Cancer* (INCa, National cancer institute). The IRFC-FC was developed in 2009 to ensure effective coordination of cancer care provision throughout the Franche-Comté region. Prior to 2009, there was only one referral center, and eight peripheral hospitals, all providing cancer care, and all totally independent, with each having their own organization. In the peripheral centers, patients were managed entirely at the discretion of local physicians, who were not necessarily specialized in oncology. Since 2009, the IRFC-FC has introduced a new system for the delivery of cancer care, with uniform cancer management practices throughout the region. It is a top-down, regional organization, with oncologists from the reference centre travelling to each peripheral center on a regular basis to offer their expertise (i.e. a mobile oncology team). This mobile oncology team works in collaboration with local physicians, who remain the primary contacts for the patients on-site. This type of organization presents several advantages. Firstly, the national cancer plan requires that a multidisciplinary committee be consulted to facilitate collective decisions on care [3]. With the IRFC-FC, each individual treatment strategy can be decided on by a multidisciplinary team. Secondly, patients can be treated near their home, yet benefitting from the same expertise as that offered at the reference center through the local availability of the mobile oncology team. Thirdly, a regional data management system has been created in order to access patient data throughout the region. This dedicated software (“Dossier Communicant Cancer”) is available to physicians and contains all individual data about cancer patients. The data are available in each center (peripheral or reference centres) regardless of where the patient is being treated. In addition, a regional application for chemotherapy prescription has been developed, based on a common guide for chemotherapy management (“Bonnes Pratiques de Chimiothérapie” Software) in order to harmonize management throughout the region. In the context of the shortage of oncologists, the mobile oncology team has made it possible to maintain cancer care services in the eight peripheral hospitals, with the same level of medical expertise as that provided in the reference center.

Although we expect the new regional oncology service to improve the quality of health care by making treatment more readily accessible to patients, we cannot exclude that upheavals in health care organization may also negatively affect quality of care. Therefore, the impact of this new regional organization needs to be carefully examined. Breast cancer is a pathology that is amenable to assessment of the impact on quality of care, because it is the most common disease in women worldwide. In 2010, the global incidence of breast cancer was nearly 1,643,000 cases, and there were about 425,000 breast cancer-related deaths worldwide, making it the leading cause of death for women [4]. We chose early breast cancer because it is very common, and curable. Using a quality approach, this study aimed to assess the impact of the new regional cancer care organization on medical management and on the costs of treating early stage breast cancer.

## Methods

Patients aged 18 years or older, with a primary breast cancer managed at the reference centre or in either of two peripheral hospitals were included. Patients were excluded if they had carcinoma *in situ* or metastatic breast cancer. We also excluded patients who refused consent, or missing patients and pregnant women. The “before” study period was from January 2007 to September 2007, and the “after” study was conducted between January 2010 and September 2010. Each patient was followed up until they met the primary endpoint (up to 01 January 2013) or died, whichever occurred first. In view of the major differences in management of patients with Her2 over-expression versus those without Her2 over-expression, and to obtain a homogeneous population, only patients without Her2 over-expression were included. All patients had testing for Her2 in both 2007 and 2010. This test is performed systematically in the French healthcare system, since its results influence the treatment strategy. In the “before” period, patients were managed by the local team (i.e. oncologists at the reference or peripheral centre). In the “after” period, all patients treated at the peripheral centres were managed by the mobile oncology team from the reference centre, in collaboration with local physicians.

Patients were identified from the medical information system (*PMSI – programme de médicalisation des systèmes d’information*) of each hospital. The PMSI is a database of standardized codes used throughout France for classifying disease. Cases were identified using the International Classification of Diseases, 10^th^ revision, diagnosis code C50 (malignant neoplasm of the breast). Administrative, clinical, biological and therapeutic data were collected from medical records, from anti-cancer drug prescription software and from the database of the regional cancer registry.

The primary outcome was the quality of patient management, as assessed by three criteria:

– Time to treatment, defined as the time interval between the first surgery and adjuvant chemotherapy, if any. Although the optimal time to initiate adjuvant therapy is uncertain, French authorities have defined delayed treatment as an interval between surgery and adjuvant therapy exceeding 12 weeks [5-7];

– Dose intensity in adjuvant chemotherapy, defined as the amount of drug delivered per unit time, expressed as mg/m^2^/week relative to the chosen standard. Full compliance was defined as actual delivery of between 85% and 115% of the theoretical amount [8-10];

– Appropriateness of therapeutic strategy, particularly the use of adjuvant chemotherapy.

The secondary outcome was 24-month progression-free survival (PFS), defined as the time between initial diagnosis and recurrence or relapse, second cancer, or death, whichever occurred first. Prognostic factors that are significantly associated with 24-month PFS can be identified by univariate analysis. One such factor is the ten-year risk, as estimated by the online tool “Adjuvant!” online. “Adjuvant!” is an online decision-making tool that estimates the risk that an individual patient will develop recurrent disease and/or die within ten years without systemic therapy (hormone therapy or chemotherapy). It can also estimate the reduction in these risks afforded by therapy (hormone therapy or chemotherapy, alone or combined). The estimates are based on established and validated factors such as age, menopausal status, estrogen receptor status, and number of axillary lymph nodes involved. In this study, we entered all data on risk factors and therapy for each patient, to obtain an estimation of the risk of recurrence (assuming no systemic therapy is given), which was then used to classify patients by ten-year risk. Adjuvant Online was not used to make decisions on therapy in this study. Several studies have validated the use of this software, and it has been used to validate other prediction models [11-13].

Lastly, we also conducted an economic analysis of patient management in our study. This retrospective economic study extends from diagnosis to progression or death. The perspective used for our study was that of French public healthcare system, and took into account the costs related to drug acquisition, hospital care for chemotherapy administration, and any hospital costs related to toxicity and transport. Indeed, all chemotherapy administration and toxicities were managed in the hospital setting. In our model, we considered only direct medical costs. Indirect costs reflecting the loss of occupational activity were not taken into account. Doses of anti-cancer drugs were given in accordance with recommendations, and were accounted for based on prices in 2010. We calculated the cost of hospitalization per day using data issued by the French public diagnosis related groups system (DRG), which does not include expensive drugs. In accordance with national pricing, we considered one session of chemotherapy to cost 385.77 Euro. Transport costs were calculated based on the distance between the patient’s home and the hospital. Patients had to be transferred to the hospital in order to receive chemotherapy. Reimbursement of transports costs in the French healthcare system varies depending on where the patient lives, and the distance travelled. Reimbursement comprises a fixed sum ranging from 11.48 Euro to 12.08 Euro, plus 0.85 Euro/kilometre as and from the fourth kilometre of distance travelled (reimbursement basis for one round trip from home to hospital, prices valid as of 1 February 2013).

The results are presented for the reference centre and for the two peripheral centres combined. Quantitative and qualitative variables were compared by using the chi-2 test or Fisher’s exact test, and the Wilcoxon signed rank test respectively. The average costs associated with the “before” group and the “after” group were compared using Student’s t test. 24-month PFS was evaluated by the Kaplan-Meier method. Comparison of local-recurrence-free survival between groups was performed using the log-rank test by univariate analysis. Multivariate Cox regression analysis was used to estimate predictors of shorter survival. All p-values were two-sided; confidence intervals were at the 95% level. Statistical analysis was performed using SAS version 9.1 (SAS Institute Inc., Cary, NC, USA).

The protocol was approved by the local ethics committee (*Comité d'Ethique Clinique du CHU de Besançon*).

## Results

**Figure 1:**
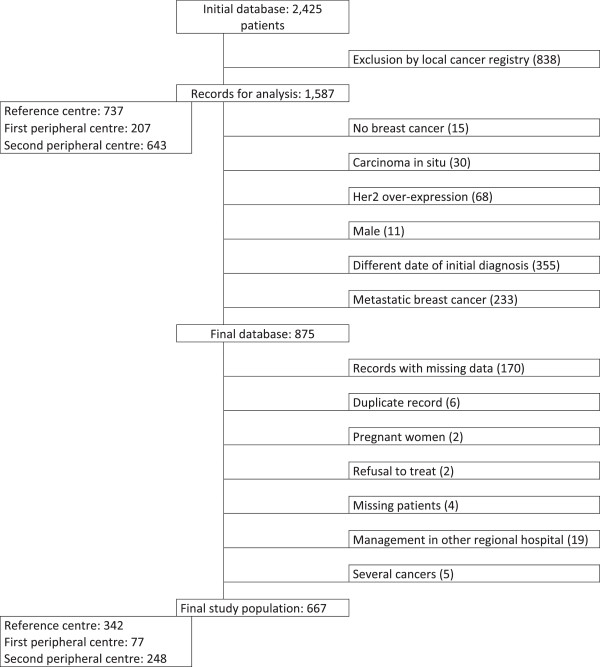
Flowchart of the study population.

Analysis of the hospital medical information systems yielded 1,587 records for analysis from the cancer registry, of whom 875 patients met the eligibility criteria for the study. After analysis of the medical records, 667 patients were included in the study (342 patients in the reference centre, and respectively 77 and 248 in the two peripheral centres); 306 corresponding to the “before” period (149 in the reference centre, 157 in the peripheral centres), and 361 to the “after” period (193 in the reference centre, 168 in the peripheral centres). A flowchart of the study population is presented in Figure 1. The patient profiles were generally comparable between the two periods. However, we observed an increase in the rates of estrogen receptor- negative tumors (10.9% in 2007 *versus* 13.7% in 2010) and SBR grade 3 tumors (14.2% versus 21.4% in 2010 respectively) in the reference centre. The percentage of patients with a risk of ten-year progression >40% (based on the Adjuvant! Online score) was not significantly different between periods (30.7% in 2007 versus 32.2% in 2010) (Table 1).

**Table 1:** Characteristics of the study population, for the “before” and “after” periods, in the reference centre and in the two peripheral centres

	**Overall**			**Reference centre**			**Peripheral centres**		
	**Before**	**After**	**p**	**Before**	**After**	**p**	**Before**	**After**	**p**
**Number**	306	361		149	193		157	168	
**Age (year)**	61.5 ± 12.5	62.3 ± 13.6	**0.31**	60.0 ± 11.9	60.4 ± 14.1	**0.68**	62.9 ± 12.9	64.1 ± 12.9	**0.30**
	61 [31–88]	62 [24–89]		61 [31–87]	61 [26–88]		62 [31–88]	64 [24–89]	
**Hormone status**									
Estrogen receptors			**0.40**			**0.51**			**0.04**
Positive	264 (86.8)	318 (89.1)		131 (89.1)	164 (86.3)		133 (84.7)	154 (92.2)	
Negative	40 (13.2)	39 (10.9)		16 (10.9)	26 (13.7)		24 (15.3)	13 (7.8)	
Progesterone receptors			**0.06**			**0.27**			**0.14**
Positive	230 (75.7)	292 (81.8)		113 (76.9)	156 (82.1)		117 (74.5)	136 (81.5)	
Negative	74 (24.3)	65 (18.2)		34 (23.1)	34 (17.9)		40 (25.5)	31 (18.6)	
All negative receptors			**0.39**			**0.61**			**0.03**
Yes	38 (12.5)	36 (10.1)		15 (10.2)	24 (12.6)		23 (14.7)	12 (7.2)	
No	266 (87.5)	321 (89.9)		132 (89.8)	166 (87.4)		134 (85.4)	155 (92.8)	
**SBR grade**			**0.43**			**0.05**			**0.62**
1	84 (28.3)	112 (31.8)		40 (28.4)	64 (34.2)		44 (28.2)	48 (29.1)	
2	162 (54.6)	174 (49.4)		81 (57.5)	83 (44.4)		81 (51.9)	91 (55.1)	
3	51 (17.2)	66 (18.8)		20 (14.2)	40 (21.4)		31 (19.9)	26 (15.8)	
**Tumor size (mm)**	21.1 ± 16.0	20.8 ± 15.6	**0.97**	20.2 ± 15.2	21.4 ± 15.9	**0.39**	21.8 ± 16.8	20.0 ± 15.3	**0.32**
	16 [1–82]	15 [1–90]		15 [1–82]	16 [1–90]		17 [1–80]	15 [3–90]	
**TNM classification**									
T (mm)			**0.92**			**0.24**			**0.46**
0 to 19	189 (63.6)	232 (65.0)		92 (63.0)	122 (63.9)		97 (64.2)	110 (66.3)	
20 to 9	87 (29.3)	101 (28.3)		46 (31.5)	54 (28.3)		41 (27.1)	47 (28.3)	
≥ 50	14 (4.7)	18 (5.0)		5 (3.4)	14 (7.3)		9 (6.0)	4 (2.4)	
All size with extension	7 (2.4)	6 (1.7)		3 (2.1)	1 (0.5)		4 (2.7)	5 (3.0)	
N			**0.05**			**0.03**			**0.62**
0	195 (62.3)	244 (69.1)		95 (63.8)	134 (70.9)		100 (64.9)	110 (67.1)	
1 to 3	81 (27.7)	90 (25.5)		41 (27.5)	45 (23.8)		40 (26.0)	45 (27.4)	
4 to 9	25 (9.4)	13 (3.7)		13 (8.7)	6 (3.2)		12 (7.8)	7 (4.3)	
≥ 10	2 (0.6)	6 (1.7)		0 (0.0)	4 (2.1)		2 (1.3)	2 (1.2)	
**Ten-year risk of progression based on AdjuvantOnline score (%)**			**0.92**			**0.37**			**0.69**
≤ 20	82 (29.6)	94 (29.4)		29 (21.8)	45 (26.0)		53 (36.8)	49 (33.3)	
]20-40]	110 (39.7)	123 (38.4)		62 (46.6)	67 (38.7)		48 (33.3)	56 (38.1)	
> 40	85 (30.7)	103 (32.2)		42 (31.6)	61 (35.3)		43 (29.9)	42 (28.6)	

Table 2 shows the adjuvant therapeutic strategies used to treat breast cancer in the study. No differences were observed as regards radiotherapy and hormone treatment rates, which were about 90 and 80% respectively. Almost all patients were treated by surgery (100.0% in the reference centre and 99.4% in the peripheral centres). We observed two major changes in the management of local breast cancer between periods. The rate of use of chemotherapy decreased, albeit without reaching statistical significance, and the use of taxane-based adjuvant therapy increased significantly by 38% overall.

**Table 2:** Type and quality of cancer services for the “before” and “after” periods, in the reference centre and in the two peripheral centres

	**Overall**			**Reference centre**			**Peripheral centres**		
	**Before**	**After**	**p**	**Before**	**After**	**p**	**Before**	**After**	**p**
**Description**									
Surgery			**1.00**			**1.00**			**0.96**
Yes	305 (99.7)	360 (99.7)		149 (100.0)	193 (100.0)		156 (99.4)	167 (99.4)	
No	1 (0.3)	1 (0.3)		0 (0.0)	0 (0.0)		1 (0.6)	1 (0.6)	
Chemotherapy			**0.07**			**0.45**			**0.04**
Yes	151 (49.3)	152 (42.2)		79 (53.0)	94 (48.7)		72 (45.9)	58 (34.7)	
No	155 (50.7)	208 (57.8)		70 (46.9)	99 (51.3)		85 (54.1)	109 (65.3)	
Exposition to taxane			**<0.0001**			**<0.0001**			**<0.0001**
Yes	90 (59.6)	149 (98.0)		51 (64.6)	93 (98.9)		39 (54.2)	56 (96.6)	
No	61 (40.4)	3 (2.0)		28 (35.4)	1 (1.1)		33 (45.8)	2 (3.4)	
Hormone therapy			**0.84**			**0.51**			**0.63**
Yes	251 (82.3)	293 (81.4)		118 (79.7)	148 (76.7)		133 (84.7)	145 (86.8)	
No	54 (17.7)	67 (18.6)		30 (20.3)	45 (23.3)		24 (15.3)	22 (13.2)	
Radiotherapy			**0.59**			**0.84**			**0.35**
Yes	281 (91.8)	327 (90.6)		136 (91.3)	178 (92.2)		145 (92.4)	149 (88.7)	
No	25 (8.2)	34 (9.4)		13 (8.7)	15 (7.8)		12 (7.6)	19 (11.3)	
**Delayed treatment (>12 weeks)**			**0.81**			**0.85**			**0.93**
No	302 (98.7)	357 (98.9)		148 (99.3)	192 (99.5)		154 (98.1)	165 (98.2)	
Yes	4 (1.3)	4 (1.1)		1 (0.7)	1 (0.5)		3 (1.9)	3 (1.8)	
**Time between surgery and adjuvant chemotherapy (weeks)**	6.0 ± 3.0	5.6 ± 3.6	**0.11**	5.1 ± 2.2	5.3 ± 3.7	**0.87**	6.7 ± 3.4	6.0 ± 3.4	**0.17**
	4.9 [1.0-20.4]	4.9 [0.3-31.7]		4.6 [1.6-15.4]	4.6 [0.3-31.7]		6.6 [1.0-20.4]	5.3 [1.1-17.1]	
**Dose-dense**			**0.22**			**0.60**			**0.32**
Yes	126 (86.3)	113 (91.1)		70 (90.9)	69 (93.2)		56 (81.2)	44 (88.0)	
No	20 (13.7)	11 (8.9)		7 (9.1)	5 (6.8)		13 (18.8)	6 (12.0)	
**24-month progression free survival (%)**	96.8	95.6	**0.79**	95.2	96.7	**0.68**	96.6	94.0	**0.93**

Table 2 also reports the quality of cancer care. The time between surgery and initiation of chemotherapy decreased non-significantly (6.0 ± 3.0 in 2007 *versus* 5.6 ± 3.6 in 2010, p = 0.11). According to French authorities, treatment is considered to be delayed when the interval between surgery and adjuvant chemotherapy exceeds twelve weeks. The percentage of patients treated with the appropriate dose-intensity increased, albeit non-significantly (86.3% *versus* 91.1%, p = 0.22). An additional 7% of patients received the appropriate dose-intensity in the peripheral centres during the second period as compared to the first period.

The new regional infrastructure for cancer care provision did not impact on 24-month PFS, which remained approximately the same between periods, at 96.8% before and 95.6% after (p = 0.79) (Table 2).

Univariate analysis of the prognostic factors associated with 24-month PFS showed that women with hormonal receptors (estrogen or progesterone receptors) were about 10% less likely to progress within 24 months following breast cancer diagnosis compared to the others (respectively p <0.01and p <0.001). Other prognostic factors were associated with improved PFS at 24 months were low SBR grade (p = 0.02), small tumor size (p = 0.0001), and absence of lymph node involvement (p < 0.0001). Dose-dense treatment, and timely treatment did not have a significant effect on 24-month PFS. The place of treatment did not influence the 24-month PFS (p = 0.63), suggesting that there was no centre effect (Table 3).

**Table 3:** Univariate analysis of prognostic factors associated with 24-month progression-free survival

**Variable**	**N**	**Fail**	**24-month progression-free survival (%)**	**p**
**Age (years)**				0.38
< 50	113	11	92.7	
[50–74]	395	28	97.1	
≥ 75	110	9	96.4	
**Hormonal status**				
Estrogen receptor				<0.01
Positive	539	37	97.3	
Negative	73	10	85.9	
Progesterone receptor				<0.001
Positive	482	29	97.8	
Negative	130	18	89.2	
**SBR grade**				0.02
1	186	8	98.0	
2	309	25	96.6	
3	107	13	91.3	
**TNM classification**				
T (mm)				0.0001
<20	388	19	97.9	
≥ 20	217	26	92.7	
N				<0.0001
0	405	18	98.2	
≥ 1	203	28	93.0	
Period				0.71
Before	287	32	96.8	
After	331	16	95.6	
Centre				0.63
Reference centre	305	25	96.1	
Peripheral centres	313	23	95.3	

Table 4 displays the results of the economic evaluation. The overall cost of managing local breast cancer was €3,524 ± 4,242 for the “before” period versus €3,441 ± 4,239 for the “after” period (p = 0.53) (Table 4). The average cost of chemotherapy, among only those patients receiving chemotherapy, was €3,422 for the first period versus €4,457 for the second period (p < 0.001).

**Table 4:** Economic evaluation

**Variable**	**Overall**			**Reference centre**			**Peripheral centres**		
	**Before**	**After**	**p**	**Before**	**After**	**p**	**Before**	**After**	**p**
**CHEMOTHERAPY**									
Hospitalization costs to receive chemotherapy	1,287 ± 1,416	1,172 ± 1,556	**0.33**	1,382 ± 1,407	1,304 ± 1,536	**0.62**	1,198 ± 1,424	1,020 ± 1,569	**0.26**
	0 [0 – 7,204]	0 [0 – 7,204]		2,275 [0–6,445]	0 [0–7,204]		0 [0–7,204]	0 [0–6,445]	
Chemotherapy drugs	1,698 ± 2,463	1,877 ± 2,435	**0.53**	1,766 ± 2,556	2,173 ± 2,494	**0.85**	1,634 ± 2,377	1,535 ± 2,325	**0.22**
	0 [0 – 10,521]	0 [0 – 7,614]		163 [0–7,554]	0 [0–7,614]		0 [0–10,521]	0 [0–6,309]	
Transport to receive chemotherapy	551 ± 366	253 ± 433	**0.20**	319 ± 434	325 ± 491	**0.59**	187 ± 275	170 ± 338	**0.10**
	0 [0 – 1,868]	0 [0 – 2,701]		195 [0–1,839]	0 [0–2,701]		0 [0–1,868]	0 [0 – 2,094]	
Overall (all patients)	3,236 ± 3,753	3,301 ± 4,080	**0.73**	3,466 ± 3,812	3,803 ± 4,154	**0.69**	3,018 ± 3,694	2,726 ± 3,927	**0.31**
	0 [0 – 14,219]	3,301 [0 – 13,483]		2,870 [0–11,305]	0 [0–13,483]		0 [0–14,219]	0 [0–10,402]	
**INTERCURRENT HOSPITALIZATIONS**									
Hospitalization	281 ± 1,311	136 ± 589	**0.29**	169 ± 940	95 ± 784	**0.67**	389 ± 1,581	183 ± 938	**0.38**
	0 [0 – 13,725]	0 [0 – 9,297]		0 [0–6,617]	0 [0–9,297]		0 [0–13,725]	0 [0–7,282]	
Transport to be hospitalized	6 ± 40	3 ± 19	**0.30**	3 ± 18	3 ± 18	**0.69**	10 ± 53	4 ± 20	**0.38**
	0 [0 – 470]	0 [0 – 206]		0 [0–150]	0 [0–168]		0 [0–470]	0 [0–206]	
Overall	288 ± 1,347	139 ± 872	**0.29**	172 ± 956	100 ± 794	**0.67**	398 ± 1,626	187 ± 954	**0.38**
	0 [0 – 14,195]	0 [0 – 9,344]		0 [0–6,767]	0 [0–9,344]		0 [0–14,195]	0 [0–7,356]	
**OVERALL COST OF MANAGEMENT**	3,524 ± 4,242	3,441 ± 4,239	**0.53**	3,638 ± 4,074	3,901 ± 4,236	**0.77**	3,416 ± 4,406	2,913 ± 4,193	**0.20**
	1,065 [0 – 21,673]	0 [0 – 15,316]		2,893 [0–17,176]	0 [0–15,316]		0 [0–21,674]	0 [0–14,067]	

## Discussion

France is confronted with a shortage of medical oncologists. This problem creates many disparities in cancer management. Given that a growing number of regions are deprived of physicians, they are currently reconsidering ways of making healthcare available where it is needed. Access to oncologists varies considerably from one region to another, suggesting that breast cancer management is not optimal. The heterogeneity of care structures and medical practices brings about health inequalities [14]. In an attempt to deal with this problem, cancer networks have been created across the country. The aim of these networks is to offer patients equitable and good quality cancer services. Moreover, the organization of cancer networks seems to favor standardized practices, based on research data [15]. In this context, a new organization was put in place in our region to promote more widespread healthcare service, with equal access for all patients, regardless of their geographical location. The regional impact of this new organization from a quality perspective was assessed before and after its implementation.

The two populations studied present similar characteristics, except for the number of lymph nodes involved at diagnosis (N0 on TNM classification). In the “after” period, we observed more patients without lymph nodes (69.1%). This result can be explained by the fact that systematic screening allowed us to detect breast cancer earlier, as previously reported by other authors [16-18]. In a German study, almost 18% of all breast cancer cases were identified through a screening examination [19]. Benson *et al.* showed that the incidence of small (<1 cm) invasive breast cancers without axillary lymph node involvement has been increasing because of the wider use of mammographic screening [20]. However, although the N0 rate increased, this does not necessarily mean that study patients are at lower risk of progression. Indeed, the percentage of patients with a risk of ten-year progression >40% was identical in both periods.

The new organization of care in our region did not change the profile of patients treated in the peripheral centres. These centres can offer oncology treatment to patients near their place of residence, since access to necessary healthcare facilities has been maintained or improved. However, high-risk patients, whose condition was likely to deteriorate, tended to be treated mainly in the reference centre during the second period, as reflected by the increase in the rates of negative estrogen receptor tumors and SBR grade 3 tumors observed in the reference centre.

One of the feared consequences of creating a new regional healthcare delivery structure was that patient management might be delayed due to the mandatory multidisciplinary meetings. It was unclear whether mobile team management would delay treatment, because the patients were further away from the reference centre, or whether the quality of patient management might be compromised. However, our results show that these fears may have been unfounded, as management did not appear to be negatively affected by the introduction of a new structure for cancer delivery.

Although the results are not significant, efficient coordination tends to improve quality criteria by favouring faster breast cancer management, with a reduced time between surgery and adjuvant chemotherapy. It also allows patients to be treated with the appropriate dose-density. Time to treatment in our study was comparable to data from the national cancer institute (INCA), at 5.6 ± 3.6 weeks versus 5.9 ± 2.1 weeks in 2010 [21]. By contrast, the rate of delayed treatment was far lower in our study, with only 1.1% experiencing a delay before treatment, versus 42.2% in the national study. For the most part, these results can be explained by successful multidisciplinary collaboration among professionals and by local community-based management.

The new organization had no effect on 24-month PFS. Follow-up in our study was not long enough to assess patient prognosis (two years). Indeed, Ng *et al.* reported that, despite the existence of a correlation between 2-year PFS and 5-year overall survival, the correlation was not strong enough to be used as a predictor [22].

At an economic level, this study did not estimate the loss of occupational activity and related social contributions due to breast cancer, which might be substantial in this disease. Indeed, the economic impact of breast cancer is high [23-25]. The overall cost of managing local breast cancer was not significantly modified by the new organization, estimated at around €3,500. In the new healthcare structure, patients can benefit from similar quality cancer services at a similar cost. The use of taxanes led to a significant increase in the cost of chemotherapy between the two periods among patients who were treated with chemotherapy. However, the number of hospital admissions for complications tended to decrease, which subsequently reduced the overall cost. Finally, the new organization made it possible for patients to benefit from the addition of taxanes to the standard chemotherapy regimen, while limiting the overall cost. Moreover, travel costs to the reference centre can be avoided with the new set-up, and patients can be treated more comfortably in peripheral centres near their homes. Despite the shortage of oncologists, physicians manage to limit transport costs. Thus, we can suppose that the quality of life of these patients was improved with reduced travel time and cost, but further studies are warranted to confirm this hypothesis.

This study has some limitations. The major limitation is the before-and-after design, which precludes any conclusions as to whether changes in care are due to the implementation of a new regional organization for healthcare delivery. Secondly, does not assess patient satisfaction. Moreover, another major limitation is the change in practices between study periods. Between 2007 and 2010, the methods for treating patients with local breast cancer were optimized. Fewer patients received chemotherapy (7% decrease), but the chemotherapy used was more intensive, with taxane molecules. This observation is not the result of implementing a new regional organization for cancer care delivery. The greater use of taxanes over the past few years has been described in the literature, with a number of trials addressing the benefit of adding a taxane (paclitaxel or docetaxel) to an anthracycline-based adjuvant chemotherapy regimen [26-30]. However, under the new regional structure for cancer care delivery, management is now standardized throughout the region of Franche-Comté and in line with national and international recommendations.

## Conclusions

Despite a shortage of oncologists, the re-organization of cancer care delivery implemented in our region made it possible to maintain local, community-based treatment, without economic consequences. This type of organization offers cancer services of similar or even better quality, with no modification of 24-month PFS in early breast cancer. Other studies are warranted to assess the impact of this organization on quality of life, because patients can be treated near their homes and receive comparable, high-quality healthcare throughout the region.

## Abbreviations

PFS: Progression-free survival; IRFC-FC: Institut régional fédératif du cancer en Franche-Comté; INCa: Institut national du cancer; PMSI: Programme de médicalisation des systèmes d’information; DRG: Diagnosed-related group.

## Competing interests

The authors declare that they have no competing interests.

## Authors’ contributions

All authors participated in the writing of the paper. Aline Voidey drafted the manuscript. Anne-Sophie Woronoff, Xavier Pivot, Gilles Nallet, Francis Schwetterle, Laurent Cals, Samuel Limat commented on revisions to the manuscript. All authors read and approved the final manuscript.

## Pre-publication history

The pre-publication history for this paper can be accessed here:

http://www.biomedcentral.com/1472-6963/14/326/prepub
